# Nanobody-based CD38-specific heavy chain antibodies induce killing of multiple myeloma and other hematological malignancies

**DOI:** 10.7150/thno.38533

**Published:** 2020-02-03

**Authors:** Levin Schriewer, Kerstin Schütze, Katharina Petry, Julia Hambach, William Fumey, Julia Koenigsdorf, Natalie Baum, Stephan Menzel, Björn Rissiek, Kristoffer Riecken, Boris Fehse, Jana Larissa Röckendorf, Joanna Schmid, Birte Albrecht, Hans Pinnschmidt, Francis Ayuk, Nicolaus Kröger, Mascha Binder, Gunter Schuch, Timon Hansen, Friedrich Haag, Gerhard Adam, Friedrich Koch-Nolte, Peter Bannas

**Affiliations:** 1 Department of Diagnostic and Interventional Radiology and Nuclear medicine, University Medical Center, Hamburg-Eppendorf, Germany.; 2 Institute of Immunology, University Medical Center, Hamburg-Eppendorf, Germany.; 3 Department of Neurology, University Medical Center, Hamburg-Eppendorf, Germany.; 4 Research Department Cell and Gene Therapy, University Medical Center, Hamburg-Eppendorf, Germany.; 5 Institute of Medical Biometry and Epidemiology, University Medical Center, Hamburg-Eppendorf, Germany.; 6 Department of Stem Cell Transplantation, University Medical Center, Hamburg-Eppendorf, Germany.; 7 Department of Oncology and Hematology, University Medical Center, Hamburg-Eppendorf, Germany.; 8 Department of Haematology and Oncology, University Hospital Halle, Halle, Germany.; 9 Hematology and Oncology Center Altona (HOPA), Hamburg, Germany.; Nanobody® is a trademark of Ablynx. In this paper we use nanobody as the generic term for the recombinant VHH domain of a llama heavy chain antibody.

## Abstract

**Rationale**: CD38 is a target for the therapy of multiple myeloma (MM) with monoclonal antibodies such as daratumumab and isatuximab. Since MM patients exhibit a high rate of relapse, the development of new biologics targeting alternative CD38 epitopes is desirable. The discovery of single-domain antibodies (nanobodies) has opened the way for a new generation of antitumor therapeutics. We report the generation of nanobody-based humanized IgG1 heavy chain antibodies (hcAbs) with a high specificity and affinity that recognize three different and non-overlapping epitopes of CD38 and compare their cytotoxicity against CD38-expressing hematological cancer cells *in vitro*, *ex vivo and in vivo*.

**Methods**: We generated three humanized hcAbs (WF211-hcAb, MU1067-hcAb, JK36-hcAb) that recognize three different non-overlapping epitopes (E1, E2, E3) of CD38 by fusion of llama-derived nanobodies to the hinge- and Fc-domains of human IgG1. WF211-hcAb shares the binding epitope E1 with daratumumab. We compared the capacity of these CD38-specific hcAbs and daratumumab to induce CDC and ADCC in CD38-expressing tumor cell lines *in vitro* and in patient MM cells* ex vivo* as well as effects on xenograft tumor growth and survival* in vivo*.

**Results**: CD38-specific heavy chain antibodies (WF211-hcAb, MU1067-hcAb, JK36-hcAb) potently induced antibody-dependent cellular cytotoxicity (ADCC) in CD38-expressing tumor cell lines and in primary patient MM cells, but only little if any complement-dependent cytotoxicity (CDC). *In vivo,* CD38-specific heavy chain antibodies significantly reduced the growth of systemic lymphomas and prolonged survival of tumor bearing SCID mice.

**Conclusions**: CD38-specific nanobody-based humanized IgG1 heavy chain antibodies mediate cytotoxicity against CD38-expressing hematological cancer cells *in vitro*, *ex vivo and in vivo*. These promising results of our study indicate that CD38-specific hcAbs warrant further clinical development as therapeutics for multiple myeloma and other hematological malignancies.

## Introduction

Multiple myeloma (MM) is a malignant plasma cell disorder with an incidence of 4-5 per 100,000 persons per year, causing 1% of all cancer-induced deaths 1, 2. MM is characterized by bone, renal, hematological, and infectious complications due to accumulation of clonal plasma cells in the bone marrow and pathogenic antibody production 3. Survival of MM patients has improved substantially with new drug classes such as proteasome inhibitors and immunomodulatory drugs when combined with autologous stem cell transplantation 4. Despite this progress, the large majority of MM patients relapses and eventually dies from refractory disease. Moreover, current treatments are associated with severe side effects 5, 6. This highlights the need for new, effective treatment options with higher specificity and fewer side effects 7-10. Monoclonal antibodies targeting specific cell surface proteins represent an important new class of agents that may meet these needs 11-13.

The glycoprotein CD38 represents a particularly attractive target in MM as it is highly expressed on malignant plasma cells in all stages of the disease 13, 14. Moreover, CD38 is overexpressed in the majority of acute lymphoblastic leukemia cases, in some acute myeloid leukemia cases, in non-Hodgkin's lymphoma, and in a subset of patients with chronic lymphocytic leukemia 15. At the same time, CD38 is expressed only at low levels on mature lymphocytes and non-hematopoietic tissues. This expression pattern results in a favorable side-effect profile of CD38-targeting antibodies. Indeed, clinical studies have shown a marked activity of such antibodies in MM, studies in other hematological malignancies are ongoing 15. CD38-specific antibodies may play a role in the treatment of diseases beyond hematological malignancies, including solid tumors and antibody-mediated autoimmune diseases 15, 16.

Most advanced in development is daratumumab, a monoclonal anti-CD38 IgG1 antibody generated by hybridoma technology after immunization of transgenic mice 17. Daratumumab has single-agent activity and a limited toxicity profile, allowing favorable combination therapies with existing as well as emerging therapies 18. Indeed, daratumumab has shown promising anti-myeloma activity in two late-stage clinical studies (GEN501 and SIRIUS) 7, 19. Accordingly, daratumumab was approved by the US Food and Drug Administration for patients with MM who have received ≥3 prior lines of therapy 20.

Daratumumab kills MM cells via different mechanisms including complement-dependent cytotoxicity (CDC) and antibody-dependent cellular cytotoxicity (ADCC) 17. It has been proposed that these therapeutic effects are related to the specific epitope that is recognized by daratumumab on CD38 17. Binding at this unique epitope might position the Fc portion in an orientation that facilitates formation of IgG hexamers and activation of the complement cascade 21. However, the unique epitope required for daratumumab binding raises the question of whether this might be a point of vulnerability for drug resistance 18. Moreover, anti-idiotype antibodies could also neutralize the biological activity of daratumumab. To address this emerging limitation, alternative CD38-specific antibody constructs are required.

The discovery of single-domain antibodies (nanobodies, VHHs, 18 kDa) has opened the way for a new generation of antitumor therapeutics 22-30. We generated 22 distinct families of llama-derived CD38-specific nanobodies and characterized their binding epitopes 31, 32. We also generated humanized heavy chain antibodies (hcAbs, 80 kDa) by genetic fusion of CD38-specific nanobodies to the hinge- and Fc-domains of human IgG1 and characterized their CDC capacity *in vitro* 33. Here, we compared the capacity of three CD38-specific hcAbs (WF211-hcAb, MU1067-hcAb, JK36-hcAb) that recognize three different epitopes of CD38 (E1, E2, E3) and of daratumumab (epitope E1) to induce CDC and ADCC in CD38-expressing tumor cell lines *in vitro* and in patient MM cells* ex vivo* as well as effects on xenograft tumor growth and survival* in vivo*. Our results underscore the potential of hcAbs for therapy of CD38-expressing hematological malignancies.

## Materials and Methods

### CD38-specific conventional and heavy chain antibodies

Daratumumab (Darzalex) was purchased from Janssen-Cilag, Neuss, Germany, to be used as positive control in our killing assays.

Llama-derived CD38-specific nanobodies WF211, MU1067, and JK36 and the (negative) control nanobody l-15 were generated as described previously 31. Nanobodies (18 kDa) are monovalent single domain antibody fragments derived from the heavy chain IgG antibodies naturally occurring in camelids 23, 34. Nanobodies correspond to the variable domain (VHH) of these heavy chain antibodies. Their robust, soluble single domain format renders nanobodies amenable for genetic fusion to the hinge- and Fc-domains of other antibody isotypes 25, 35. The resulting bivalent chimeric llama/human heavy chain antibodies (hcAbs) acquire the capacity to induce Fc-mediated effector functions (e.g CDC, ADCC) at about half the size of a conventional monoclonal antibody (80 vs. 150 kDa) 35.

**Figure 1:**
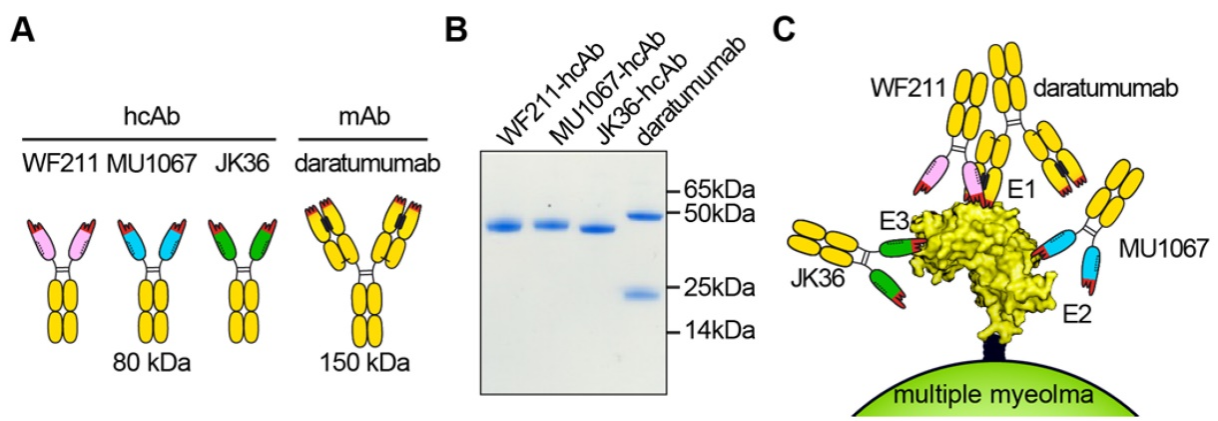
** Structure, purity, and binding epitopes of CD38-specific antibody constructs. (A)** Scheme of heavy chain antibodies (80 kDa). The single-domain antibody fragments (nanobodies) are indicated in pink (WF211), blue (MU1067), and green (JK36). The hinge and Fc-domains of human IgG1 are indicated in black and yellow, respectively. The three CDR loops of the antigen-binding paratope are indicated in red. Conventional antibody daratumumab (150 kDa) is indicated in yellow (heavy chains and light chains). Hydrophobic patches at the interface between the VH and VL domains are indicated in black. The corresponding hydrophilic patch in the nanobodies is indicated by dashed lines. **(B)** Coomassie-staining of an SDS-PAGE under reducing conditions with CD38-specific heavy chain antibodies WF211-hcAb, MU1067-hcAb, JK36-hcAb and daratumumab verifies the purity and integrity of the constructs used in this study. Note that daratumumab consists of two heavy chains (50 kDa) and two light chains (25 kDa). The hcAbs consist of two identical heavy chains (42 kDa). Each lane contains 2 µg of the indicated construct.** (C)** The three hcAbs recognize three distinct epitopes of CD38. WF211-hcAb recognizes epitope 1 (E1), MU1067-hcAb recognizes epitope 2 (E2), and JK36-hcAb recognizes epitope 3 (E3). The binding epitope E1 of WF211-hcAb overlaps with the epitope of daratumumab.

The heavy chain antibodies WF211-hcAb, MU1067-hcAb, and JK36-hcAb **(Figure 1A)** were generated by subcloning the coding region of the nanobodies upstream of the coding region for the hinge- and Fc-domains of human IgG1 in the pCSE2.5 vector (kindly provided by Thomas Schirrmann, Braunschweig, Germany) 33. The Fc-domains of our hcAbs thus are the same (human IgG1) as that of daratumumab 17. L-15 is a nanobody directed against the enzymatic subunit CDTa of *Clostridium difficile* 36, the corresponding heavy chain antibody (l-15-hcAb) served as the isotype control in all experiments.

Recombinant hcAbs were expressed in transiently transfected HEK-6E cells cultivated in serum-free medium. Six days post transfection, supernatants were harvested and cleared by centrifugation. HcAbs were purified by affinity chromatography using protein G-sepharose. Purity of antibody constructs was assessed by SDS-PAGE and Coomassie Brilliant Blue staining.

### Cell lines

Daudi and CA46 lymphoma cell lines as well as the LP-1 myeloma cell line were obtained from the German Collection of Microorganisms and Cell Culture (DSMZ, Braunschweig, Germany).

Cell lines (CA46-luc, Daudi-luc, LP-1-luc) stably expressing the luc2 variant of *Photinus pyralis* luciferase (Promega, Madison, WI) under control of the spleen-focus-forming virus U3 region (SFFV promoter) were generated by lentiviral transduction. The vector was cloned by inserting the luc2 cDNA (Addgene plasmid #24337) in front of the internal ribosome entry site of the HIV-1 derived, 3^rd^ generation, self-inactivating lentiviral vector LeGO-iG2-Puro+ co-expressing the fluorescent marker eGFP linked to a puromycin resistance by a 2A-sequence 37. Production of lentiviral particles was performed as described 38. Transduction of target cells was carried out in a 24-well plate with 50.000 cells in 500 µL medium per well by addition of 300 µL viral-particle containing supernatant in presence of 8 µg/mL polybrene and subsequent spin-inoculation for 1 hour at 1000×g and 25°C. Transduced cells were selected in culture medium containing 1 µg/mL puromycin. Stably transduced cells were FACS sorted (FACS Aria III, BD Biosciences, Heidelberg, Germany) based on eGFP expression.

Mouse Yac-1 lymphoma cells were transfected with an expression vector for human CD38 by electroporation (250 mV, 960 μF) using 3 μg DNA/10^7^ cells in 400 μL RPMI and a Gene pulser (Bio-Rad GmbH, Munich, Germany). Stable transfectants (Yac-1-CD38) were obtained by selection in medium supplemented with blasticidin (10 µg/mL). Cells were subcloned by limiting dilution, and clones were analyzed for CD38 expression levels by flow cytometry. Cell lines were cultured in RPMI 1640 medium (Gibco, Life Technologies, Paisley, UK) supplemented with 2 mM sodium pyruvate (Gibco), 2 mM L-glutamine (Gibco) and 10% (v/v) fetal calf serum (Gibco).

NK-92, a human NK cell line, was obtained from DSMZ. NK-92 cells stably co-expressing GFP and human CD16 were obtained by retroviral transduction using the pSF91 retroviral vector 39. The sequence for CD16, i.e. the ectodomain of Fc*γ*RIII fused to the transmembrane and cytosolic domains of FcεRI was kindly provided by Béatrice Clémenceau, Nantes, France 40. The CD38 gene was inactivated in these cells using CRISPR/Cas9 technology (sc-401117-NIC, Santa Cruz Biotechnology). NK-92-CD16-GFP cells and NK-92-CD16-GFP-CD38ko cells were FACS-sorted and grown in alpha MEM culture medium (Gibco) supplemented with 12.5% FCS (Gibco), 12.5% horse serum (Gibco), 100 IU/mL IL-2 (Proleukin, Novartis, Nürnberg, Germany), and 2 mM L-glutamine (Gibco).

### Binding affinities and epitope mapping of CD38-specific reagents

Binding specificities of hcAbs were assessed by staining of untransfected CD38-negative parental Yac-1 cells and their counterparts stably transfected with human CD38 with hcAbs WF211-hcAb, MU1067-hcAb, JK36-hcAb, isotype control (l-15-hcAb), or daratumumab. Binding was detected with a PE-conjugated donkey anti-human IgG-specific secondary antibody (Dianova, Hamburg, Germany). Control stainings were performed with the PE-conjugated secondary antibody alone. Cell-associated fluorescence was determined by flow cytometry.

Binding affinities of hcAbs were assessed by incubation of Yac1-CD38 cells with serial dilutions of antibodies. Cells were washed and incubated with PE-conjugated donkey anti-human IgG (Dianova, Hamburg, Germany). Cell-associated fluorescence was determined by flow cytometry.

The relative dissociation rates of Alexa^647^-conjugated monovalent nanobodies and bivalent antibodies from cell surface CD38 were assessed by incubation of Yac1-CD38 cells with excess (100 nM) of fluorochrome-conjugated nanobodies, heavy chain antibodies or daratumumab for 30 min at 4°C. Cells were washed three times and then monitored for loss of cell-associated fluorescence over time at RT. An aliquot of CD38-expressing Yac-1 cells that had been labeled with the cell-tracking dye eFluor 450 was added at t = 0 as a sink for dissociated nanobodies or antibodies. Cell-associated fluorescence was determined by flow cytometry.

Epitope mapping was performed by incubation of CD38-expressing LP-1 lymphoma cells with a saturating concentration (100 nM) of unconjugated hcAbs WF211-hcAb, MU1067-hcAb, JK36-hcAb, or daratumumab for 30 min at 4°C before addition of Alexa^647^-conjugated CD38-specific hcAbs or daratumumab (25 nM). Cells were further incubated for 30 min at 4°C, washed twice, and analyzed by flow cytometry. Percentage of cross-blockade was then calculated from mean fluorescence intensities (MFI) as follows:


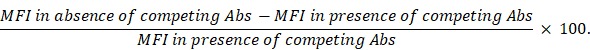


### CD38 expression analyses

Untransduced and GFP/luciferase-transduced CA46, Daudi, and LP-1 cells were incubated in PBS at 1x10^6^ cells per assay with 1 µg/mL Alexa^647^-conjugated MU1067-hcAb for 1 hour at 4°C. Samples were washed and analyzed by flow cytometry for expression of CD38 and GFP.

### CDC of tumor cell lines

CA46-luc, Daudi-luc, or LP-1-luc cells were incubated with WF211-hcAb, MU1067-hcAb, JK36-hcAb, isotype control (l-15-hcAb), or daratumumab as positive control. The incubation was performed in the presence of 15% pooled human serum as source of complement for 60 min at 37°C. Heat inactivated (30 min at 56°C) serum was used as control to verify complement dependency. CDC was quantified by flow cytometric measurement of propidium iodide (PI) uptake. Percentage of lysed cells was defined as percentage of PI-positive cells.

### ADCC of tumor cell lines

CA46-luc, Daudi-luc and LP-1-luc cells were incubated with serial dilutions of hcAbs or daratumumab. NK-92-CD16 cells were added as effector cells at an effector to target ratio [E:T] of 3:1.

Peripheral blood mononuclear cells (PBMCs) containing primary NK cells were obtained from buffy coats from healthy donors by Ficoll-Paque density gradient centrifugation and subsequent depletion of erythrocytes using lysis buffer (NH_4_Cl + KHCO_3_ + EDTA). To activate NK cells, PBMCs were incubated overnight in alpha MEM culture medium supplemented with 12.5% FCS, 12.5% horse serum, 100 IU/mL IL-2 (Proleukin, Novartis, Nürnberg, Germany), and 2 mM L-glutamine (Gibco). These cells were added as effector cells at an effector to target ratio [E:T] of 30:1.

Cells were then co-incubated for 4 h at 37°C. Synthetic D-luciferin (Biosynth, Staad, Switzerland) was added as substrate (150 µg/mL) for 20 min and bioluminescence (BLI) was measured with a microplate reader (Victor³, Perkin Elmer, Boston, USA). Percentage of lysed cells was calculated as follows 41:


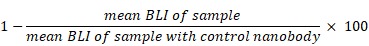


### Mouse xenograft tumor model

Tumor xenograft experiments were conducted using female, 12-14 week old Fox Chase SCID mice (CB17/Icr-*Prkdc^scid^*/IcrIcoCrl) obtained from Charles River Laboratories (Sulzfeld, Germany). Mice weighed 22 ± 2.5 g (range 20 to 25 g). Water and food were provided ad libitum. Mice were checked daily for signs of discomfort and for general appearance. Body weight was measured three times a week. Experiments were performed in accordance with international guidelines on the ethical use of animals and were approved by the local animal welfare commission (TVA 17/13).

4x10^6^ CA46-luc cells were intravenously injected in 200 µL saline solution. Mice were randomly grouped (n = 7 per group) and treated by weekly i.p. injections of 200 µL saline solution containing 50 µg (~2 mg/kg) of CD38-specific hcAbs WF211-hcAb, MU1067-hcAb, JK36-hcAb, the isotype control l-15-hcAb, or daratumumab. Weekly treatments started on day 7 after inoculation, i.e. at a time point where tumors were detected in all inoculated mice. Mice were treated 6 times until day 42 after tumor inoculation.

*In vivo* imaging was performed at weekly intervals starting one week after xenograft inoculation directly before the first antibody treatment. Mice were anesthetized with isofluorane and intraperitoneally injected with synthetic D-luciferin (6 mg in 200µL PBS). After 15 minutes, mice were positioned in the imaging chamber of the small-animal imaging system (IVIS-200, PerkinElmer, Boston, MA, USA). Luminescence was measured by counting photons emitted during an exposure period of 1 min. Under illumination, black-and-white images were made for anatomical reference. Rectangular regions of interest (ROIs) were placed around individual mice for quantitative analyses. Total flux [photons/sec] was determined with Living Image 4.2 software (PerkinElmer).

Animals were euthanized when turning moribund according to pre-defined criteria (weight loss >20%, loss of ability to ambulate, labored respiration, or inability to drink or feed) in order to avoid animal suffering.

### CDC and ADCC of primary MM cells

Fresh primary MM cells were obtained from bone marrow aspirates after IRB-approved consent was obtained from all patients. Experiments were performed in accordance with the ethical standards of the responsible committee on human experimentation and with the Helsinki Declaration. The study was approved by the local IRB committee (PV5505). Bone marrow mononuclear cells (BM-MNCs) were prepared by Ficoll-Paque density gradient centrifugation of bone marrow aspirates and subsequent depletion of remaining erythrocytes using red blood cell lysis buffer (NH_4_Cl + KHCO_3_ + EDTA). Patient characteristics are provided in **Table 1.**

For CDC assays, BM-MNCs were incubated in PBS+0.2% BSA and 100nM CD38-specific hcAbs, isotype control (l-15-hcAb), or daratumumab and 12.5% pooled human serum as a source of complement for 90 min.

For ADCC assays, BM-MNCs were incubated in alpha MEM and 100nM CD38-specific hcAbs, isotype control, or daratumumab and NK92 cells stably transduced with CD16 and GFP at an effector to target ratio [E:T] of 10:1 for 2.5 h.

For both assays, cells were then stained with a panel of antibodies (CD38, CD45, CD138/CD229/CD55/CD59, CD269/CD319/CD56, CD19) and analyzed via flow cytometry. Staining of CD38 was achieved with Alexa^647^-conjugated Nbs that bind independently of the hcAb/mAb used for CDC/ADCC: JK36^647^ or MU523^647^ for daratumumab and WF211-hcAb, MU523^647^ for JK36-hcAb, and JK36^647^ or WF211^647^ for MU1067-hcAb. An FSC threshold was set to exclude debris while including the population of small CD19+ B cells. In case of ADCC, GFP-expressing NK-92 cells were excluded by gating. MM cells were identified by high co-expression of CD38 and CD138. Numbers of MM cells were determined using CountBright absolute counting beads (Invitrogen). Percentage of surviving MM cells was calculated as follows: MM cell number of αCD38 hcAb or mAb treated sample / MM cell number of control hcAb treated sample x 100.

### Statistical Analysis

For ADCC of tumor cell lines, a linear regression on the basis of a one-phase decay was performed and a one-way ANOVA was used to determine significant differences between the three hcAbs and daratumumab.

For xenograft tumors, a two-way ANOVA followed by a Bonferroni post hoc test was used to determine significant differences of light emissions between treatment groups. Survival curves were analyzed using Kaplan-Meier plots and strata compared using the log-rank test (GraphPad Prism).

For CDC and ADCC of primary MM cells, the number of MM cells per mL transformed to the natural logarithm was considered the dependent variable for statistical analysis using a mixed model approach (SPSS routine GENLINMIXED). A normal data distribution was assumed, and an identity link function was applied. Antibody-construct and cytotoxicity-assay were considered fixed effect variables in the model, patient-by-cytotoxicity-assay as random effect and antibody-construct within patient-by-cytotoxicity-assay as repeated measures. Model-estimated marginal means of constructs were compared pairwise within cytotoxicity-assays, with Sidak-adjusting of the alpha error for multiple comparisons. All tests were two-sided and a p-value <0.05 was considered statistically significant. Statistical analysis was performed using SPSS v. 25.

## Results

### CD38-specific hcAbs show higher binding avidities than the respective monovalent nanobodies

We produced and purified three nanobody-based humanized heavy chain antibodies by genetically fusing nanobodies WF211, MU1067, JK36 to the hinge- and Fc-domains of human IgG1 **(Figure 1B)**. Competition binding studies with WF211-hcAb, MU1067-hcAb, and JK36-hcAb on Yac-1-CD38 cells confirmed specific binding to three distinct epitopes E1, E2, and E3 on the extracellular domain of CD38. Blocking experiments further revealed that the binding epitope of WF211-hcAb (E1) overlaps with the epitope of the conventional CD38-specific human IgG1 antibody daratumumab **(Figure 1C, Table 2)**.

**Figure 2:**
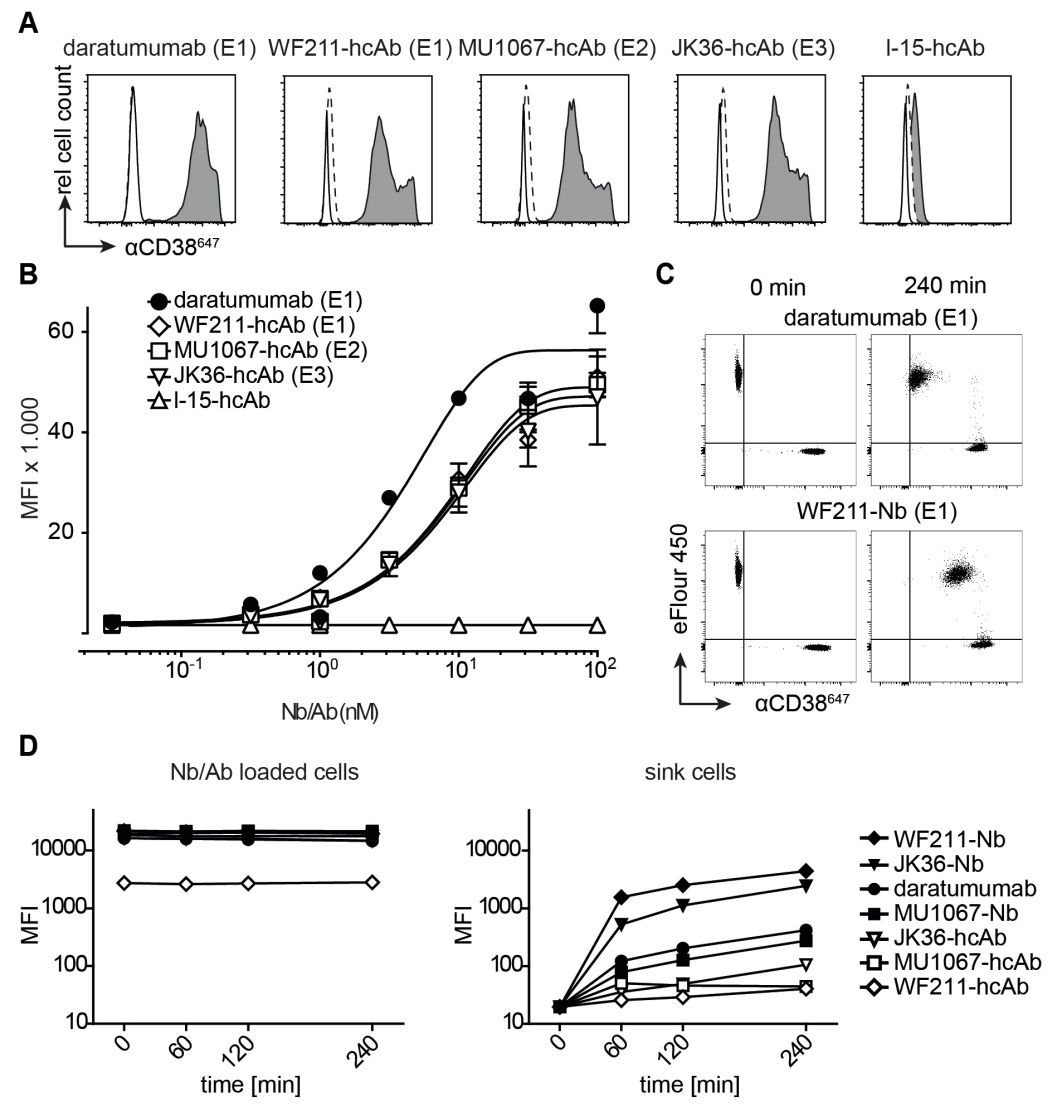
** Binding of CD38-specific hcAbs to lymphoma cells. (A)** Untransfected CD38-negative parental Yac-1 cells (open histograms) and their counterparts stably transfected with human CD38 (grey histograms) were stained with CD38-specific hcAbs WF211-hcAb, MU1067-hcAb, JK36-hcAb, isotype control (l-15-hcAb), or daratumumab. Binding was detected with a PE-conjugated human IgG-specific secondary antibody. Control stainings were performed with the PE-conjugated secondary antibody alone (dashed lines). Epitope specificities of CD38-specific antibodies are given in parentheses. **(B)** Titration analysis of the binding of CD38-specific hcAbs to CD38-transfected Yac-1 cells. Cells were incubated with serial dilutions of CD38-specific hcAbs, isotype control (l-15-hcAb), or daratumumab. Binding was detected with a PE-conjugated human IgG-specific secondary antibody. Data represent mean ± SD from three independent experiments. MFI, mean fluorescence intensity.** (C)** Representative dot plots and **(D)** changes in mean fluorescence intensity over time of the dissociation of fluorochrome-conjugated monovalent nanobodies and bivalent hcAbs from CD38-transfected Yac-1 cells. Cells were incubated with excess (100 nM) Alexa^647^-conjugated nanobodies, hcAbs or daratumumab for 30 min at 4°C. Cells were washed three times and then monitored for loss of cell-associated fluorescence over time at RT. An aliquot of CD38-expressing Yac-1 cells that had been labeled with the cell-tracking dye eFluor 450 was added at t = 0 as a sink for the dissociated antibody constructs.

Assessment of binding affinities using serial titration analyses of unconjugated antibodies revealed good and comparable binding of all three CD38-specific hcAbs, regardless of epitope specificity, to CD38-expressing Yac-1 cells **(Figure 2A)**. EC50 values of hcAbs were 0.90 nM for WF211-hcAb, 0.91 nM for MU1067-hcAb, and 0.89 nM for JK36-hcAb. Daratumumab showed slightly stronger binding with an EC50 of 0.45 nM. Isotype control heavy chain antibody l-15-hcAb did not show any detectable binding.

To assess the suitability of fluorochrome-conjugated nanobodies and heavy chain antibodies for flow cytometry, we analyzed their relative dissociation rates from CD38-expressing Yac-1 cells (**Figure 2B**). Untreated, eFluor labeled cells were used as a "sink" for the dissociated antibodies. The results indicate that bivalent heavy chain antibody have increased avidity compared to the respective monovalent nanobody.

### CD38-specific hcAbs do not effectively induce CDC of tumor cell lines *in vitro*

**Figure 3:**
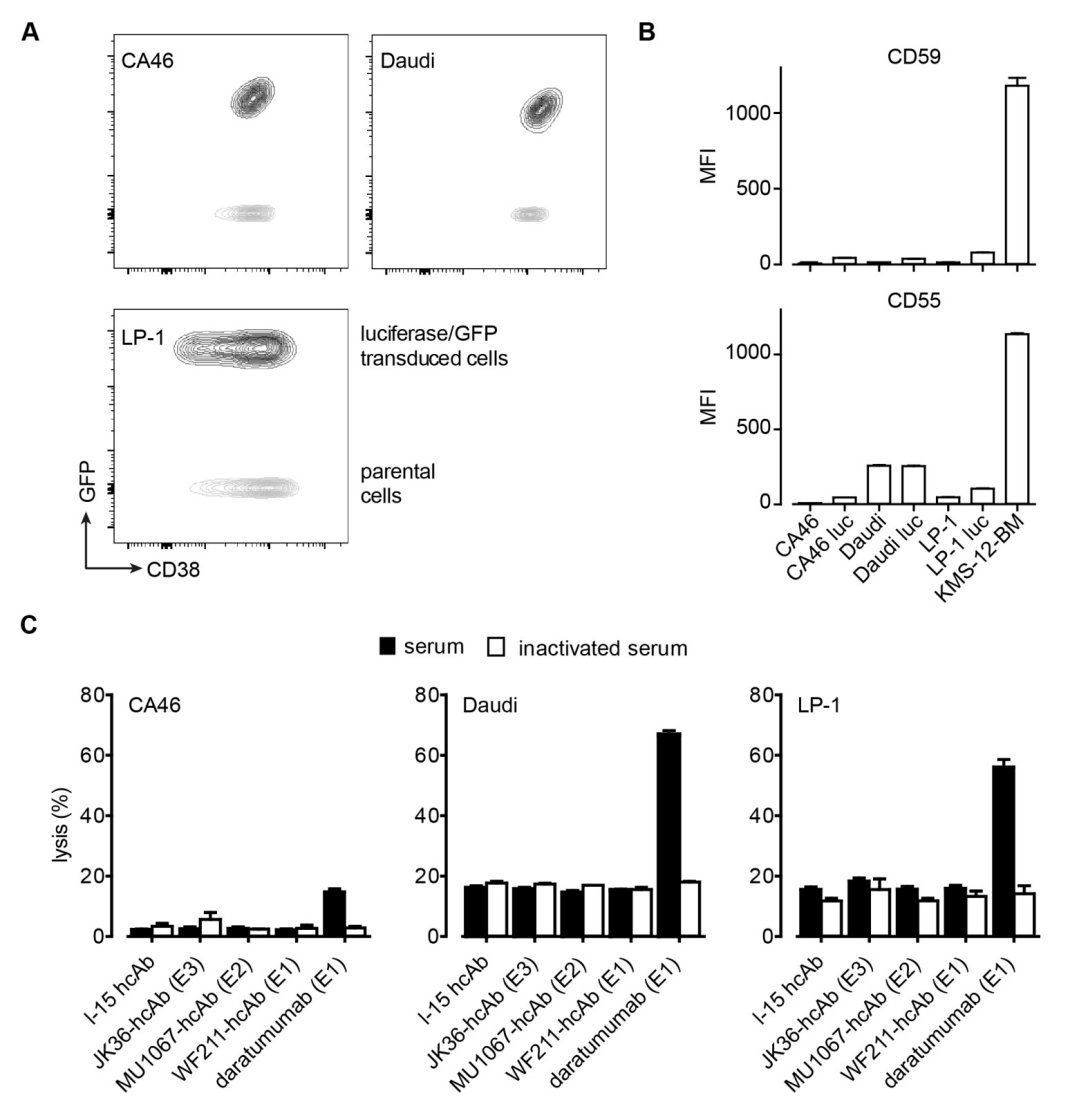
** Cell surface expression of CD38, CD59, CD55 and CDC induction by CD38-specific hcAbs. (A)** Cell surface expression of CD38 by human lymphoma cell lines (CA46, Daudi, LP-1) before and after transduction with GFP/luciferase. Non-transduced and GFP/luciferase-transduced cells were stained with Alexa^647^-conjugated MU1067-hcAb and analyzed by flow cytometry. Dot plots were overlaid for direct comparison of CD38 expression levels by non-transduced parental cells (GFP-) and GFP/luciferase-transduced cells (GFP+).** (B)** Cell surface expression of CD55 and CD59 by CA46, Daudi, and LP-1 cells before and after transduction with GFP/luciferase. **(C)** CA46, Daudi, and LP-1 cells were incubated for 60 min at 37°C with CD38-specific heavy chain antibodies WF211-hcAb, MU1067-hcAb, JK36-hcAb, isotype control (l-15-hcAb), or daratumumab in the presence of 15% pooled human serum as source of complement. The same pooled serum pretreated for 30 min at 56°C (inactivated serum) was used as control. Cells were then stained with propidium iodide and analyzed by flow cytometry to determine the percentage of lysed cells. Epitope specificities of CD38-specific antibodies are indicated in parentheses. Data represent mean ± SD from three independent experiments.

The ability of CD38-specific hcAbs to induce complement-dependent cytotoxicity (CDC) was tested with a human multiple myeloma cell line (LP-1) and two human Burkitt's lymphoma cell lines (Daudi, CA46). These cells express moderate to high levels of CD38 **(Figure 3A)** and low to moderate levels of the complement-inactivating surface proteins CD55 and CD59 (**Figure 3B**). The three CD38-specific hcAbs induced little if any CDC in the three tested cell lines. In contrast, daratumumab induced varying degrees of complement-dependent lysis depending on the cell line **(Figure 3C)**. Daratumumab induced highest lysis (67 ± 1%) of Daudi cells, intermediate lysis (56 ± 3%) of LP-1 cells, and lowest lysis (15 ± 1%) of CA46 cells. Control experiments with inactivated serum resulted only in background levels of tumor cell lysis, confirming CDC as one mechanism of action of daratumumab. Similarly, experiments with the isotype control heavy chain antibody l-15-hcAb resulted only in background levels of tumor cell lysis, reflecting the inability of this antibody to bind to CD38.

### CD38-specific hcAbs mediate effective ADCC of tumor cell lines *in vitro*

**Figure 4:**
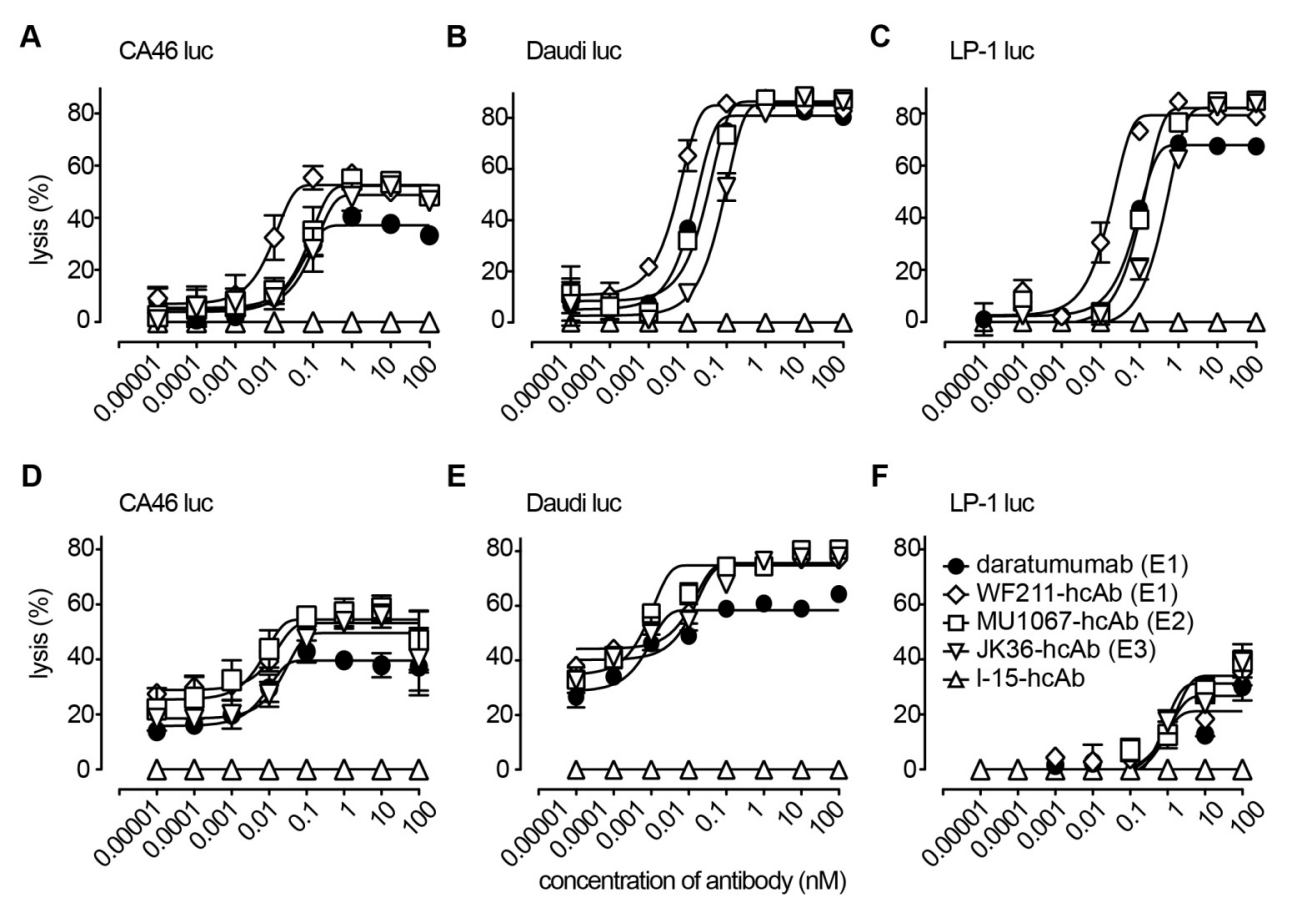
** CD38-specific hcAbs induce ADCC. (A-C)** CD16-transduced NK-92 effector cells were co-cultured for 4h at an effector to target ratio of 3:1 with luciferase (luc)-transduced lymphoma cells **(A:** CA46, **B:** Daudi, **C:** LP-1) in the presence of serial dilutions of heavy chain antibodies WF211-hcAb, MU1067-hcAb, JK36-hcAb, isotype control (l-15-hcAb), or daratumumab. **(D-F)** Primary NK effector cells were co-cultured for 4h at an effector to target ratio of 30:1 with luciferase (luc)-transduced lymphoma cells **(D:** CA46, **E:** Daudi, **F:** LP-1) in the presence of serial dilutions of hcAbs or daratumumab. Luciferin was added for 20 min at RT, and mean bioluminescence (BLI) was quantified to determine percentage of lysis. Epitope specificities of CD38-specific antibodies are indicated in parentheses. Data represent mean ± SD from three independent samples. Results are representative of three independent experiments.

The ability of CD38-specific hcAbs to induce antibody-dependent cellular cytotoxicity (ADCC) was tested with luciferase-expressing tumor cell lines LP-1, Daudi, and CA46 as targets and CD16-transduced NK-92 cells or primary NK cells as effector cells. The three hcAbs effectively induced dose-dependent lysis of all three cell lines **(Figure 4)**.

In case of NK-92 effector cells **(Figure 4A-C)**, highest maximal lysis with saturating doses of the three hcAbs (85-87%) was observed for Daudi-luc cells, without being statistically different from daratumumab (81%) (all p>0.05). In LP-1-luc cells, the maximal lysis of the three hcAbs ranged from 79-82% and was significantly higher than with daratumumab (68%) in the case of MU1067-hcAb and JK36-hcAb (both p<0.05), whereas there was no statistically significant difference between WF211-hcAb and daratumumab (p>0.05). Lowest maximal lysis of the three hcAbs was observed for CA46-luc cells (49-53%). Of note, the observed maximal lysis for all three hcAbs was significantly higher than for daratumumab (37%) (all p<0.01).

In case of primary NK effector cells **(Figure 4D-E)**, highest maximal lysis with saturating doses of the three hcAbs (75-76%) was also observed in Daudi-luc cells, being statistically significantly higher for all three hcAbs than for daratumumab (58%) (all p>0.001). In CA46-luc cells, the maximal lysis for the three hcAbs ranged from 50-55% and was significantly higher than with daratumumab (40%) in the case of MU1067-hcAb (p<0.001) and WF211-hcAb (p<0.01), whereas there was no statistically significant difference between JK36-hcAb and daratumumab (p>0.05). Lowest maximal lysis for the three hcAbs was observed for LP-1-luc cells (27-34%), without being statistically significant different from daratumumab (21%) (all p>0.05).

Isotype control heavy chain antibody l-15-hcAb did not mediate any detectable lysis, reflecting its inability to bind CD38.

### CD38-specific hcAbs inhibit tumor growth in a mouse xenograft model

**Figure 5:**
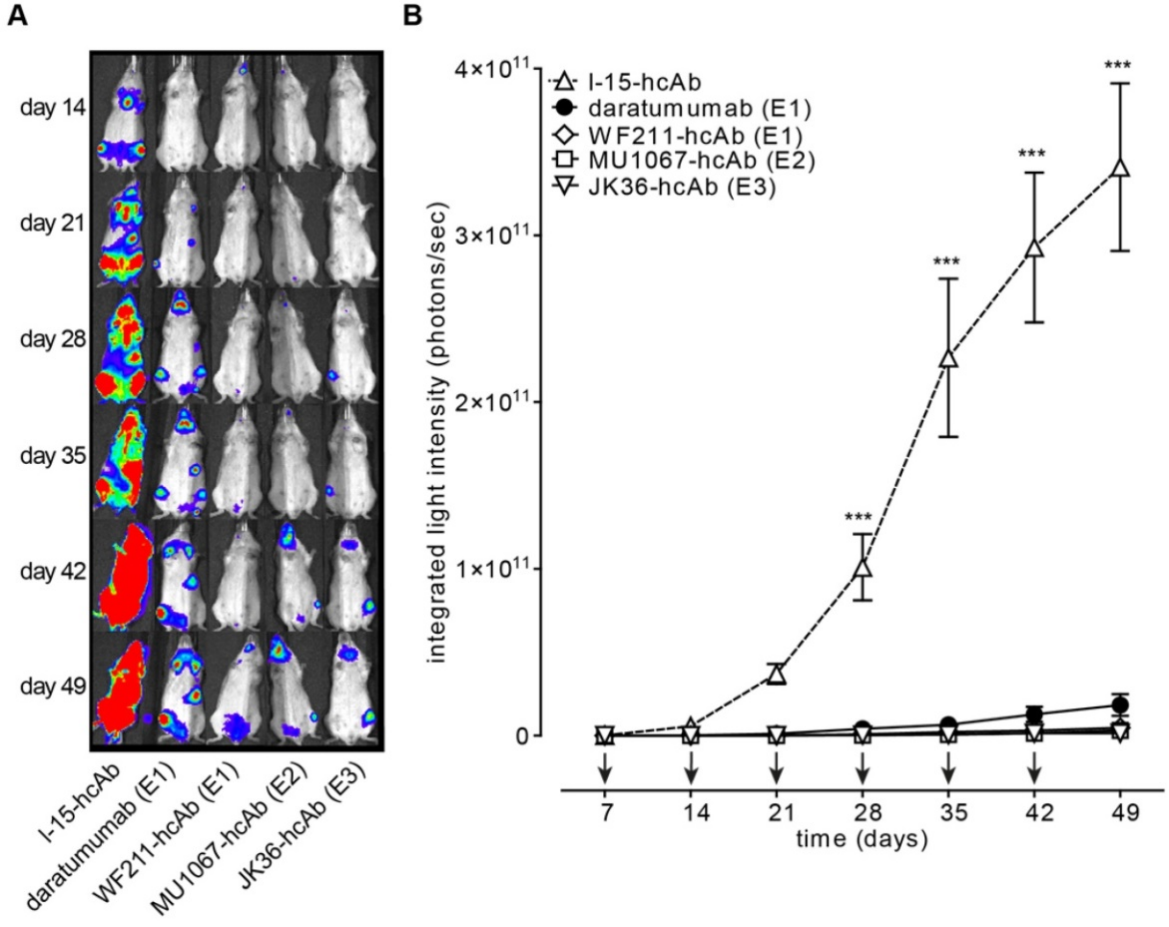
** CD38-specific hcAbs inhibit the growth of CD38-expressing CA46 tumors *in vivo*.** SCID mice (n = 7/group) were injected i.v. with luciferase-expressing CA46 cells (4 x 10^6^ cells). Starting on day 7 after tumor injection, mice received weekly i.p. injections (2 mg/kg) of CD38-specific heavy chain antibodies WF211-hcAb, MU1067-hcAb, JK36-hcAb, isotype control (l-15-hcAb), or daratumumab. **(A)** At the indicated time points after tumor-injection, bioluminescent images of mice were obtained 15 min after i.v. injection of luciferin. Signal intensities of all mice and imaging time points are equally leveled (1x10^8^ - 1x10^11^ photons/sec) to allow direct and fair visual comparison. **(B)** Light emission of tumors integrated over total body area plotted over time as a measure of tumor mass development. Arrows indicate antibody administrations. Data represent mean ± SD*.* From day 28 onward, tumor growth was significantly reduced in animals treated with hcAbs or daratumumab as compared to animals treated with isotype control (*** = p<0.001). Two-way ANOVA followed by a Bonferroni post hoc test was used for statistical analysis. Results are representative of three independent experiments. Epitope specificities of CD38-specific antibodies are indicated in parentheses.

The ability of CD38-specific hcAbs to inhibit tumor growth *in vivo* was tested in mouse xenograft experiments after systemic administration of CA46-luc cells. CA46-cells were chosen because tumor growth *in vivo* with these cells showed less variability than with Daudi-luc or LP-1-luc cells. Treatment with hcAbs or daratumumab was initiated at day 7, i.e. when tumors became detectable by luminescent imaging. The results revealed effective tumor growth inhibition *in vivo* with all three hcAbs WF211-hcAb, MU1067-hcAb, and JK36-hcAb. **Figure 5A** shows representative images of *in vivo* luminescence tumor signals over time. Animals treated with the irrelevant isotype control heavy chain antibody showed unaffected tumor growth. In contrast, treatment with CD38-specific hcAbs or with daratumumab resulted in significant inhibition of tumor cell growth as compared with isotype control treatment from day 28 (all p<0.001, as compared with isotype control treatment from day 28) **(Figure 5B)**. Administration of hcAbs showed a trend toward stronger inhibition of tumor cell growth as compared to daratumumab, however without reaching a statistically significant difference (all p>0.05).

### CD38-specific hcAbs improve survival in a mouse xenograft model

**Figure 6:**
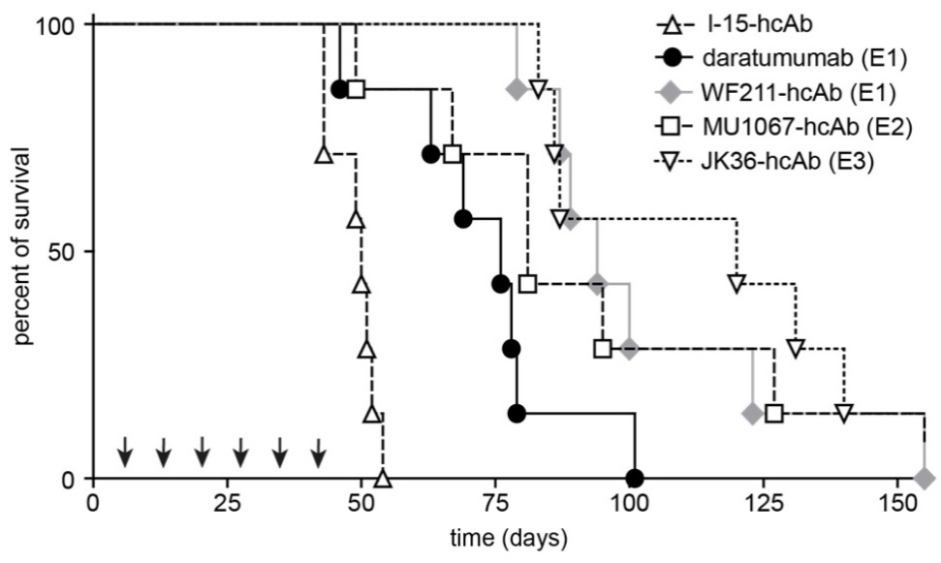
** CD38-specific hcAbs prolong the survival of mice bearing CD38-expressing CA46 tumors.** Kaplan-Meier plot of overall survival of SCID mice intravenously injected with CA46-luc cells. SCID mice (n = 7/group) received weekly i.p. treatments (2 mg/kg, arrows) with CD38-specific heavy chain antibodies WF211-hcAb, MU1067-hcAb, JK36-hcAb, isotype control (l-15-hcAb), or daratumumab as described in Fig. 5. Overall survival of mice treated with hcAbs or daratumumab was significantly longer as compared to mice receiving isotype control treatment (p≤0.0001 and p=0.002, respectively). Overall survival of mice treated with the CD38-specific hcAbs was longer than that of mice receiving daratumumab treatment. This difference was significant for JK36-hcAb (p=0.003) and WF211-hcAb (p=0.031), but not for MU1067-hcAb (p=0.110). Log rank test was used for statistical analysis. Epitope specificities of CD38-specific antibodies are indicated in parentheses. Results are representative of three independent experiments.

Mice with xenograft tumors receiving repeated treatment with any of the CD38-specific hcAbs (all p≤0.0001) or daratumumab (p=0.002) demonstrated improved overall survival compared to mice receiving control treatment **(Figure 6)**. Median survival of SCID mice receiving control treatment was 50 days after intravenous injection of CA46-luc cells, which was significantly shorter compared to 75 days of mice receiving daratumumab (p=0.002) or mice treated with any of the CD38-specific hcAbs (all p≤0.0001). The longest median survival was observed in the JK36-hcAb group (119 days) and WF211-hcAb group (92 days), which was significantly longer than median survival (75 days) of mice receiving daratumumab (p=0.003 and p=0.031, respectively). The group of mice receiving MU1067-hcAb had a median survival of 80 days, not significantly different from that of the daratumumab group (p=0.110).

### CD38-specific hcAbs induce little CDC in primary MM cells *ex vivo*

**Figure 7:**
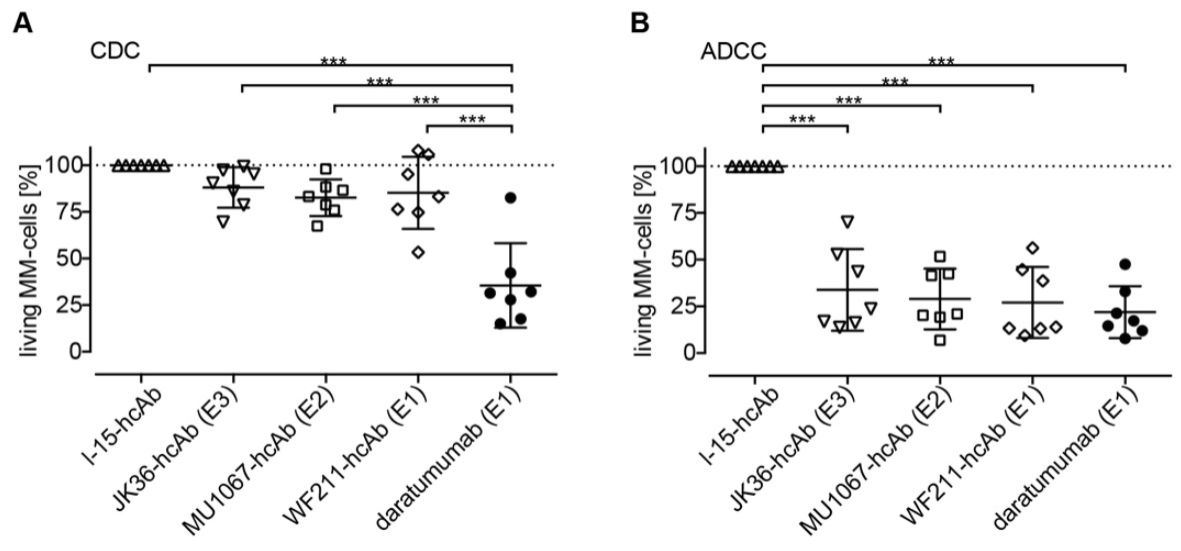
** CDC and ADCC induction by CD38-specific hcAbs of primary myeloma cells obtained from MM patients. (A)** Fresh bone barrow mononuclear cells from MM patients were incubated for 90 min with CD38-specific heavy chain antibodies WF211-hcAb, MU1067-hcAb, JK36-hcAb, isotype control (l-15-hcAb), or daratumumab in the presence of 12.5% pooled human serum as source of complement. CDC was assessed by flow cytometric quantification of the number of living MM cells.** (B)** Fresh bone marrow mononuclear cells were incubated for 2.5 h with CD16- and GFP-transduced NK-92 effector cells at an effector to target ratio of approximately 10:1 in the presence of WF211-hcAb, MU1067-hcAb, JK36-hcAb, isotype control (l-15-hcAb), or daratumumab. ADCC was assessed by flow cytometric quantification of the number of living MM cells. Data are presented as % living MM cells relative to samples treated with the isotype control. Data represent means with 95%-confidence intervals. Sidak-adjusted p-values are indicated (***= p<0.001).

The ability of CD38-specific hcAbs to induce CDC was tested on fresh bone marrow samples from MM patients. The three CD38-specific hcAbs induced little CDC of primary MM cells **(Figure 7A)**. The percentage of surviving MM cells was 88% (CI: 78-99%) for JK36-hcAb, 83% (CI: 73-92%) for MU1067-hcAb, and 85% (CI: 67-104%) for WF211-hcAb. In contrast, daratumumab induced effective CDC resulting in 36% (CI: 14-57%) of surviving cells.

### CD38-specific hcAbs mediate effective ADCC in primary MM cells *ex vivo*

The ability of CD38-specific hcAbs to induce ADCC was tested on fresh bone marrow samples from MM patients. All three hcAbs effectively induced lysis of primary MM cells **(Figure 7B)**. The percentage of surviving MM cells was 34% (CI: 14-55%) for JK36-hcAb, 29% (CI: 14-44%) for MU1067-hcAb and 27% (CI: 9-45%) for WF211-hcAb. The daratumumab-mediated ADCC was comparable resulting in 22% (CI: 9-35%) of surviving cells.

## Discussion

We demonstrated the feasibility of using CD38-specific hcAbs to efficiently kill hematological cancer cells *in vitro*, *ex vivo* and *in vivo*. Specifically, we generated nanobody-based humanized IgG1 hcAbs with a high specificity and affinity that recognize three different and non-overlapping epitopes of CD38. CD38-specific hcAbs induced potent ADCC regardless of their epitope specificity, but failed to induce substantial CDC of tumor cell lines* in vitro* or primary MM cells *ex vivo.* CD38-specific hcAbs significantly reduced tumor growth *in vivo* and significantly prolonged survival of xenograft bearing mice.

All three CD38-specific hcAbs (WF211-hcAb, MU1067-hcAb, JK36-hcAb) had high and comparable binding affinities regardless of their epitope specificity. The stronger blockade of daratumumab^647^ by MU167-hcAb than vice versa is likely explained by a higher affinity of MU1067-hcAb^647^ for CD38: in cells that have been pretreated with unlabeled daratumumab, addition of MU1067-hcAb^647^ resulted in partial displacement of daratumumab. This interpretation is consistent with the faster dissociation of daratumumab from LP-1 cells than MU1067-hcAb.

However, despite binding with high affinity to CD38, the CD38-hcAbs showed little if any capacity to induce CDC of primary MM cells, confirming previous *in vitro* results using tumor cells lines 33. Conversely, all three CD38-specific hcAbs effectively induced ADCC of tumor cell lines and primary MM cells with comparable potency to daratumumab. Interestingly, the heavy chain antibody WF211-hcAb that recognizes the same CD38 epitope (E1) as daratumumab showed the strongest ability to induce ADCC *in vitro*.

However, the observations made *in vitro* did not fully translate into the findings made in a systemic tumor xenograft mouse model* in vivo*. Despite the fact that CD38-hcAbs effectively induced ADCC but not CDC* in vitro*, all three hcAbs reduced tumor growth *in vivo* at least as effectively as daratumumab.

Previous studies have shown that CD38-targeting conventional monoclonal antibodies can mediate cytotoxicity against CD38-expressing hematological cancer cells via CDC, ADCC, antibody-dependent cellular phagocytosis (ADCP), direct induction of apoptosis, and modulation of CD38 ectoenzyme function 12, 42-44. Although different conventional antibodies target the same antigen and induce similar degrees of ADCC, marked differences in CDC capabilities were observed when comparing different CD38-specific antibodies 42. This is in line with our finding that all three CD38-specific hcAbs induce ADCC regardless of their epitope specificity, but mediate little if any CDC.

The relative contributions of CDC and ADCC to the overall therapeutic activity of monoclonal antibodies are still unknown 18. A combination of these mechanisms likely underlies the therapeutic efficacy. Our results show that CD38-specific hcAbs induce little CDC *in vitro* and *ex vivo* but mediate potent growth inhibition of systemic tumors in a mouse model. In this regard, our hcAbs have similar properties to another conventional CD38 antibody, MOR202, which does not induce CDC, but performs well in murine models, although not as well as daratumumab in clinical trials 45. Future clinical studies are needed to assess whether the ADCC effect of CD38-specific hcAbs translates into high clinical efficacy.

Our study has important potential clinical implications, particularly for patients with reduced biological activity of daratumumab. The development of neutralizing anti-idiotype antibodies may reduce the biological activity of daratumumab. Moreover, the unique epitope recognized by daratumumab could be mutated so as to prevent its binding, presenting a point of vulnerability for drug resistance 18. In such cases, the CD38-specific hcAbs described here that bind to alternative epitopes (MU1067-hcAb, JK36-hcAb) may provide alternative therapeutics. Moreover, they could be used to complement daratumumab in hematological cancer therapies.

Of note, CD38 expression levels on the surface of MM cells during daratumumab treatment are downregulated 46. Progression of MM during daratumumab therapy may occur due to the reduced CD38 levels 47. Targeting non-overlapping (MU1067-hcAb, JK36-hcAb) or overlapping CD38 epitopes (WF211-hcAb) with our nanobody-based hcAbs during this period may not be helpful and underlines the need for antibodies targeting alternative target-proteins such as BCMA and SLAMF7 on the surface of MM cells 12, 48.

Several questions could not be answered in our study and warrant further investigation: First, it will be interesting to assess the capability of our CD38-specific hcAbs to induce other effector functions, such as ADCP, direct induction of apoptosis, and modulation of CD38 ectoenzyme function. Second, binding and cytotoxicity assays involving other CD38 positive cells, including hematopoietic progenitor cells, activated immune effector cells, T regulatory cells, and endothelial cells are needed to gauge the potential therapeutic index. Third, future preclinical and clinical studies are warranted to assess the therapeutic efficacy of CD38-specific hcAbs in combination with other anti-MM agents and the efficacy in myeloma cell lines and patient cells resistant to conventional or novel therapies. Moreover, it will be of interest to explore the potential of CD38-specific hcAbs for treatment of diseases beyond hematological malignancies, including solid tumors and antibody-mediated autoimmune diseases 15, 16.

## Conclusion

In conclusion, we show that CD38-specific nanobody-based humanized IgG1 heavy chain antibodies mediate cytotoxicity against CD38-expressing hematological cancer cells *in vitro*, *ex vivo and in vivo*. These promising results of our study indicate that CD38-specific hcAbs warrant further clinical development as therapeutics for multiple myeloma and other hematological malignancies.

## Figures and Tables

**Table 1:** Patient characteristics of Multiple Myeloma patients.

Parameter	MM-patients (n=8)	
Median age, years (range)	65	(50-75)
Sex, male, n (%)	5	(62.5%)
**Serum analyses**		
Median M-Protein in IgG subtype, g/L (range)	20	(17-24)
Median serum free light chain, mg/L (range)	62	(17-8393)
**Subtype of immunoglobulins**		
IgG κ, n (%)	5	(62.5%)
IgG λ, n (%)	1	(12.5%)
κ light chain only, n (%)	1	(12.5%)
λ light chain only, n (%)	1	(12.5%)
**Bone marrow analyses**		
Median bone marrow infiltration, % (range)	23%	(10-60%)
**Previous therapy**		
No prior treatment, n (%)	6	(75%)
Prior stem cell transplantation, n (%)	2	(25%)
Autologous, n (%)	2	(25%)
Allogeneic, n (%)	0	(0%)

**Table 2:** Epitope mapping of CD38-specific hcAbs and daratumumab.

	Epitope	1	1	2	3
**Epitope**	**Name**	daratumumab^647^	WF211-hcAb^647^	MU1067-hcAb^647^	JK36-hcAb^647^
**1**	daratumumab	98*	86*	19	1
**1**	WF211-hcAb	97*	87*	22	-6
**2**	MU1067-hcAb	95*	12	98*	-3
**3**	JK36-hcAb	-2	-1	-2	97*

Numbers indicate the percentage of cross-blockade by competing antibodies. Unconjugated blocking antibodies are indicated on the left, AF647-labeled detecting antibodies are indicated on the top. Self-blockade is indicated along the diagonal in bold fond and inhibition of binding by >85% is indicated by *. Negative numbers indicate enhanced labeling of cells in the presence of the competing nanobody. Data represent mean values from three independent experiments.

## Abbreviations

ADCC: Antibody-dependent cellular cytotoxiccity
CDC: Complement dependent cytotoxicity
MM: Multiple Myeloma
